# Chemotherapy Regimens and Survival in Pancreatic Cancer—A Ten‐Year Single Centre Overview

**DOI:** 10.1002/cam4.71416

**Published:** 2025-12-02

**Authors:** Hedda Tranvik, Farima Brandt, Caroline Williamsson, Hanna Sternby

**Affiliations:** ^1^ Department of Clinical Sciences, Skåne University Hospital Lund University Lund Sweden; ^2^ Department of Surgery, Institution of Surgical Sciences Akademiska Hospital Uppsala Sweden

**Keywords:** chemotherapy, palliative, pancreas cancer, resection, survival

## Abstract

**Background:**

During the last decade chemotherapy recommendations have changed for patients with pancreatic adenocarcinoma (PDAC) as novel regimens demonstrate better survival in selected cohorts. Herein we aim to, in a regional cohort, analyse therapy changes and possible survival benefits over a 10‐year period.

**Methods:**

Patients in the southern region of Sweden diagnosed with pancreatic cancer between 2010 and 2011 (Early group) and 2018 and 2019 (Late group), were enrolled from the Swedish Pancreatic Cancer Registry. Baseline characteristics and chemotherapy regimens were obtained from medical records. Resected and palliative patients were analysed separately.

**Results:**

A total of 323 patients with PDAC were included: 81 resected and 242 palliative, 147 within the Early and 176 within the Late group. Both palliative and resected patients in the Late group were in general older with more comorbidities and more advanced disease, although few significant differences were found. The palliative patients in the Late group received more neoadjuvant, second‐line and third‐line treatment; however without impact on survival. Both groups demonstrated a clear transition towards combination therapies over the years. At 2 years after diagnosis overall survival was improved in the Late resected group with 82 versus 60%, adjusted *p* = 0.014.

**Conclusions:**

Since 2010 there has been a shift in chemotherapy treatment for pancreatic cancer patients towards the use of combination therapies. Our data demonstrates an increase in 2‐year survival for resected patients, whereas corresponding figures for the palliative patients remain unchanged.

## Graphical abstract

Pancreatic cancer patients in the group resected in 2018 and 2019 showed significantly better survival 24 months after surgery compared to those who had surgery in 2010 and 2011.

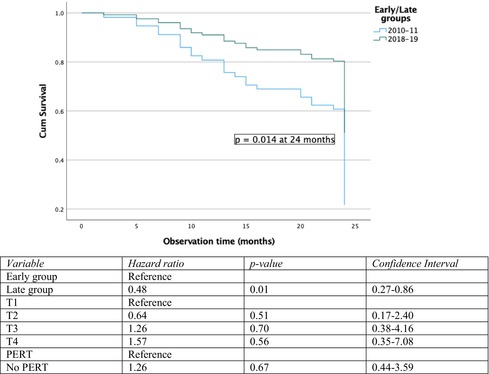

## 1 Introduction

Annually, 1900 patients are diagnosed with pancreatic adenocarcinoma (PDAC) in Sweden, corresponding to an incidence of just above 10 per 100,000 [1]. Patients commonly present at a late stage and only a minority will be subjected to treatment with a curative intent. The median 5‐year overall survival (OS) is 6% with a corresponding figure of 25% for the resected group [2]. Despite generally increasing knowledge of pancreatic cancer, the prognosis has been equally dismal over the last decades. To prolong survival, chemotherapy is the standard recommendation not only for palliative patients but also as adjuvant treatment given to resected patients [3, 4, 5]. For many years the gold standard chemotherapy for pancreatic cancer was gemcitabine in monotherapy as it had demonstrated fewer adverse effects and, in the palliative situation improved survival compared to the previous regimen with single fluorouracil (5‐FU) [6]. During the last 15 years various combination therapies have been presented showing only a slight improvement in survival [7, 8, 9, 10]. In 2011, Conroy et al. [11] demonstrated that a combination of fluorouracil, leucovorin, irinotecan and oxaliplatin (FOLFIRINOX) was, associated with an almost doubled overall survival for patients with metastatic disease. However, this treatment came at a cost of significantly higher levels of toxicity. Due to difficulties tolerating FOLFIRINOX treatment, a modified variant is often administered (mFOLFIRINOX) which is considered to be as effective as the original version but with fewer side effects [12, 13]. Second‐line therapies were generally not accepted in clinical practice until the 2010s when the results from the CONCO‐003 trial changed that approach [14].

Since 2010, pancreatic cancer care is centralised to six tertiary university hospitals in Sweden, where each hospital is a referral center for a defined region. All patients are treated according to the Swedish national guidelines for pancreatic cancer management [15]. The southern region has a population of almost 2 million people with Skåne University Hospital as the referral center for all pancreatic cancer patients. The chemotherapy recommendations within the national guidelines are partly based on the patient's performance status (PS). The scale, developed by the Eastern Cooperative Oncology Group, spans from 0 to 5, where 0 means the patient is fully active without any physical restrictions, and five represents the patient being dead [16].

– If a patient is suitable for curative treatment and undergoes surgery, adjuvant treatment for 6 months is recommended. Patients with a PS of 0–1 are recommended mFOLFIRINOX, 1–2 are recommended gemcitabine plus capecitabine (GemCap) and patients with higher PS may be given single gemcitabine [9, 10, 17].
– Neoadjuvant therapy may be considered for borderline resectable patients with a low PS, with mFOLFIRINOX as first choice and gemcitabine plus nab‐paclitaxel (GemNabP) as second choice [18]. Neoadjuvant therapy for primarily resectable patients is as yet only conducted within clinical studies [19, 20].
– Palliative treatment is recommended for patients who are assessed to benefit from it. Patients under the age of 75 with a PS of 0–1 are recommended mFOLFIRINOX or GemNabP [8, 11]. Patients with a higher PS are recommended GemCap or single gemcitabine, alternatively single 5‐FU [6, 21].
– As second line therapy, patients with progress on a gemcitabine‐based treatment are recommended mFOLFIRINOX if they have a PS of 0–1 [22]. Patients with a PS > 1 are recommended 5‐FU or capecitabine either as single therapy or in combination with oxaliplatin or irinotecan [14, 23]. Patients who progress on mFOLFIRINOX may receive single gemcitabine or GemCap.
– There is no scientific evidence regarding the efficacy of third line therapy. Therefore no general recommendations exist and treatment will only be on an individual basis.
– Radio‐chemotherapy is not standard treatment and only rarely used in selected palliative cases.

Pancreatic enzyme replacement therapy (PERT) has been demonstrated to influence survival in both resected and palliative patients [24, 25]. Still, no sharp recommendations on PERT for PDAC patients are given in the current Swedish guidelines despite the possible benefits.

To date, no studies have demonstrated to what grade current global chemotherapy recommendations have been implemented in Sweden. Herein we aim to investigate whether there, over the last decade, has been a change in given regimens for patients with PDAC and its possible impact on survival.

## 2 Methods

Data on all patients diagnosed with pancreatic adenocarcinoma from 1st of January 2010 to 31st of December 2019 in the southern Swedish region (Region Skåne) was obtained from the Swedish National Pancreatic and Periampullary Cancer registry. For comparison over time, patients diagnosed 2010–2011 (Early group) and 2018–2019 (Late group) were enrolled. For a consort diagram of patient inclusion see Figure S1. Only patients diagnosed between 2012 and 2017 were removed from the data file and no other exclusion criteria were utilised. Data on basic patient characteristics and cancer‐related information as well as chemotherapy regimens were retrieved from medical records. Comorbidities were defined as follows: heart disease (diagnosis of high blood pressure, heart failure or previous cardiac events), lung disease (asthma, chronic obstructive pulmonary disease or any other disease affecting the lungs), renal failure and diabetes. The 8th edition of the TNM system was used for tumour classification. T‐stage for the palliative group was estimated based on computer tomography assessment or other imaging modalities. The resected group was staged primarily through the post‐operative pathology report and all patients were reviewed according to the TNM 8 system. The Union for International Cancer Control (UICC) stages for resected patients are presented in Table S3. Resected and non‐resected patients were analysed separately. Follow‐up for all patients was at least 24 months; zero patients were lost during follow‐up and all reported events occurred within this time period.

Data were analysed using Chi‐2 or Fisher's Exact test for comparison of nominal groups, Student's *t*‐test for continuous and normally distributed data, and Mann Whitney *U* for continuous and non‐normally distributed data. Survival was analysed using the Cox regression technique. In the Cox regression adjustment was made for age, comorbidity, weight loss, PERT and tumour stage as well as Early versus Late group. The level of significance was set at a *p*‐value of < 0.05. All data analysis was performed using IBM SPSS Statistics 27.

Ethical approval was obtained from the national board of Ethical decisions, number 2020‐01402.

## 3 Results

A total of 323 patients, 242 palliative and 81 resected, diagnosed with pancreatic adenocarcinoma during 2010–2011 (Early group, 147 patients) and 2018–2019 (Late group, 176 patients) were included. Median follow‐up for all patients was 53 months (95% CI: 43.2–62.8).

### 3.1 Palliative Patients

#### 3.1.1 Baseline Characteristics

Of the 242 palliative patients, 110 were diagnosed in 2010–2011 and 132 in 2018–2019. Baseline characteristics of both groups are presented in Table 1. Patients in the Late group seemed generally older with more comorbidities. The distribution of locally advanced and metastatic disease was similar between the groups and no variation regarding PERT was found. When comparing TNM status, there was no change in T stage over time. For N and M status, data showed higher numbers for NX in the Early group and N1 in the Late group and similarly more M0 in the Early group and M1 in the Late group (see Table S1).

**TABLE 1:** Baseline characteristics of palliative patients.

	2010–2011	2018–2019	*p*
Total	110	132	
Age, years^a^	68 (62–75)	73 (66–77)	0.09
Sex^b^			
Female	49 (45)	68 (52)	0.28
Male	61 (56)	64 (49)	
BMI^a^	24.1 (21–27)	23.7 (21–26)	0.65
Heart disease^b^	58 (53)	77 (58)	0.38
Lung disease^b^	8 (7)	23 (17)	0.02
Diabetes^b^	25 (23)	49 (37)	0.02
Kidney failure^b^	5 (5)	7 (6)	0.60
Weight loss^b^	66 (60)	92 (70)	0.12
Locally advanced disease^b^	55 (50)	62 (47)	0.82
Metastatic disease^b^	55 (50)	70 (53)	0.74
Ca19‐9, value at diagnosis^a^	497 (116–2300)	595 (100–3478)	0.79
Jaundice upon diagnosis^b^	61 (56)	60 (46)	0.10
PERT^b^	48 (44)	57 (43)	0.94
Overall survival, months^a^	5 (3–7)	6 (4–7)	0.86
2‐year survival^b^	8 (7.3)	6 (4.5)	0.65

Abbreviations: BMI, body mass index; PERT, pancreatic exocrine replacement therapy.

^a^ Median (interquartile range).

^b^ *n* (%).

#### 3.1.2 Chemotherapy Regimens

Data on the chemotherapy regimens of the Early and Late groups of palliative patients is presented in Table 2, with a detailed therapy description in Table S2. Overall there was no difference in the number of patients receiving any form of chemotherapy. However, more patients in the Late group received neoadjuvant treatment as well as second‐line and third‐line regimens, and a larger number of the Early group had radio‐chemotherapy. Regarding the neoadjuvant regimen, the Late group received more mFOLFIRINOX (9.8% vs. 2.7%, *p* = 0.026). As first‐line treatment, the Early group more often had single gemcitabine (44.5% vs. 17.4%, *p* < 0.001) and GemCap (9.1% vs. 0.0%, *p* < 0.001), while the Late group received more combination therapies such as mFOLFIRINOX (17.4% vs. 0.9%, *p* < 0.001) and GemNabP (28.8% vs. 0.9%, *p* < 0.001). Similarly, second‐line regimens varied in distribution where both groups received the same amount of FLOX (fluorouracil + oxaliplatin). The Early group had more often GemCap (4.5% vs. 0.0%, *p* = 0.018) while the Late group received more GemNabP (8.3% vs. 0.0%, *p* = 0.002).

**TABLE 2:** Chemotherapy regimens for palliative patients.

	2010–2011	2018–2019	*p*
	*n* (%)	*n* (%)	
Any chemotherapy	63 (57)	89 (67)	0.10
Neoadjuvant treatment	4 (4)	14 (11)	0.04
Radio‐chemotherapy	11 (10)	0 (0)	< 0.001
First line treatment	62 (56)	86 (65)	0.16
Combination therapy in first line	13 (21)	63 (73)	< 0.001
Second line treatment	18 (16)	38 (29)	0.02
Third line treatment	3 (3)	14 (11)	0.02
Additional chemotherapy	0 (0)	3 (2)	0.25

#### 3.1.3 Survival

**FIGURE 1:**
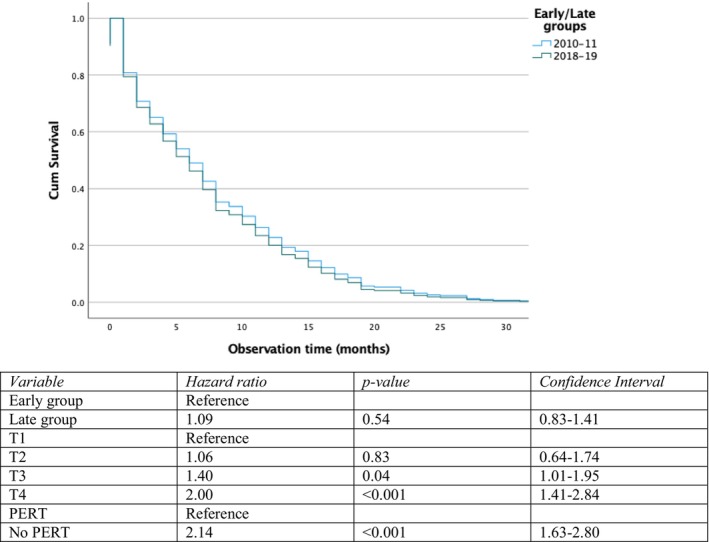
Adjusted Cox regression analysis of palliative patients.

Data on survival is presented in Table 1. No difference was found neither for median overall survival time nor 2‐year survival. In adjusted Cox regression analysis (Figure 1) survival was influenced by and adjusted for T3‐stage (hazard ratio 1.40, *p* 0.044, CI), T4‐stage (hazard ratio 2.00, *p* < 0.001, CI: 1.41–2.84) and PERT (hazard ratio 2.14, *p* < 0.001, CI: 1.63–2.80).

### 3.2 Resected Patients

#### 3.2.1 Baseline Characteristics

Baseline characteristics for resected patients are presented in Table 3. In total 81 patients were included, 37 in the Early and 44 in the Late group. Severe (Clavien‐Dindo ≥ 3) post‐operative complications were more common in the Late group. Moreover no significant differences were found; however there were more patients with heart disease in the Late group while weight loss prior to diagnosis was more frequently reported in the Early group. Cancer status according to TNM and UICC (no metastases were identified) was similar in the two time periods (Table S3).

**TABLE 3:** Baseline characteristics of resected patients.

	2010–2011	2018–2019	*p*
Total	37	44	
Age^a^	68 (62.5–72.5)	72 (63.25–76.75)	0.11
Sex^b^			
Female	16 (43.2)	22 (50)	0.65
Male	21 (56.8)	22 (50)	
BMI^a^	24.2 (21.8–26.9)	24.6 (23.1–27.4)	0.65
Comorbidity^b^	21 (56.8)	28 (63.3)	0.53
Heart disease^b^	18 (48.6)	27 (61.4)	0.25
Lung disease^b^	2 (5.4)	3 (6.8)	0.79
Diabetes^b^	11 (29.7)	15 (34.1)	0.68
Kidney failure^b^	2 (5.4)	3 (6.8)	0.79
Weight loss^b^	27 (73)	26 (59)	0.19
Ca19‐9, value at diagnosis^a^	94 (19–226)	122 (40–357)	0.14
Jaundice upon diagnosis^b^	26 (70.3)	28 (63.6)	0.53
Biliary drainage^b^	14 (66.7)	19 (59.4)	0.59
Tumour locus^b^			
Head	27 (73.0)	32 (72.7)	0.86
Body/tail	10 (27.0)	12 (27.3)	0.79
PERT^b^	34 (91.9)	38 (86.4)	0.43
Overall survival, months^a^	29 (13–98)	27 (24–41)	0.58
2‐year survival^b^	22 (59.5)	36 (81.8)	0.03

Abbreviations: BMI, body mass index; PERT, pancreatic exocrine replacement therapy.

^a^ Median (interquartile range).

^b^ *n* (%).

#### 3.2.2 Chemotherapy Regimens

The chemotherapy regimens of the resected groups are presented in Table 4 with detailed descriptions in Table S4. The vast majority of the patients in both groups received some kind of chemotherapy and no differences were found regarding neoadjuvant and adjuvant treatments. However, there was a numerical tendency towards the Late group receiving more first‐ and second‐line as well as combination therapies. Significant differences were demonstrated regarding the selection of adjuvant treatment where the Early group received more gemcitabine (81.1% vs. 22.7%, *p* < 0.001), while the Late group received more GemCap (50.0% vs. 8.1%, *p* < 0.001). In the Early group there was a tendency to more often give gemcitabine (21.6% vs. 6.8%, *p* = 0.1) and GemCap (8.1% vs. 0.0%, *p* = 0.06) as first‐line treatment, whereas the Late group to a larger extent received GemNabP (20.5% vs. 0.0%, *p* = 0.004). No significant differences were demonstrated regarding the distribution of second‐line regimens.

**TABLE 4:** Chemotherapy regimens for resected patients.

	2010–2011	2018–2019	*p*
	*n* (%)	*n* (%)	
Any chemotherapy	34 (91.9)	39 (88.6)	0.72
Neoadjuvant treatment	1 (2.7)	4 (9.1)	0.23
Adjuvant treatment	33 (89.2)	36 (81.8)	0.53
Radio‐chemotherapy	0 (0.0)	0 (0.0)	n.a.
First line treatment (palliative)	15 (40.5)	25 (56.4)	0.18
Second line treatment (palliative)	6 (16.2)	13 (29.5)	0.19
Third line treatment (palliative)	1 (2.7)	3 (6.8)	0.62
Additional chemotherapy	1 (2.7)	1 (2.3)	1.00

#### 3.2.3 Survival

Data on survival for resected patients is presented in Table 3 showing that 82% of the patients in the Late group were still alive 24 months after diagnosis compared to 60% in the early group (*p* = 0.026 in Fisher's Exact test and *p* = 0.014 in adjusted Cox regression analysis).

**FIGURE 2:**
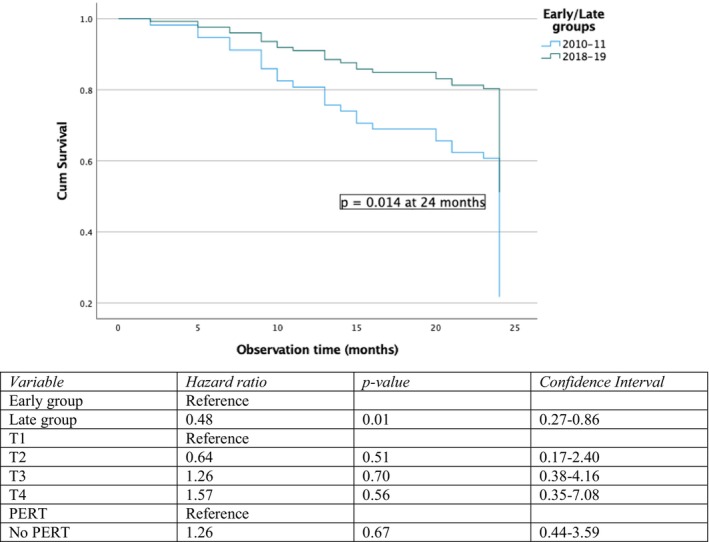
Adjusted Cox regression analysis of resected patients.

Figure 2 presents the adjusted Cox regression plot with an adjusted risk of death hazard ratio of 0.48 for the late group at 24 months after diagnosis (*p* = 0.014, CI: 0.27–0.86). No other variable influenced survival.

## 4 Discussion

The aim of this study was to review given chemotherapy regimens for pancreatic adenocarcinoma during the last 10 years in the Swedish southern region. Additionally, overall survival was investigated. Data herein demonstrate that local strategies for treatment of pancreatic cancer follow national recommendations which have changed for both the palliative and the resected patients over the last decade. Overall survival in the palliative group is still dismal, whereas the 2‐year survival rate of the resected patients diagnosed in 2018 and 2019 has improved significantly. No difference in tumour stage was found between the Early and Late groups. A possible improvement in survival is thus rather due to both the use of adjuvant combination therapies and patient selection but also multi‐agent chemotherapies as first and second line treatments [26, 27].

The collected data indicates that both palliative and resected pancreatic cancer patients of today are older and have more comorbidities compared to 10 years ago. Globally the incidence of pancreatic cancer is rising, partly due to ageing populations. Previous studies have shown that elderly PDAC patients are often undertreated as well as underrepresented in clinical trials, both resulting in a lack of managing directions [28, 29]. Recent data demonstrate that older patients, despite accompanying diseases, benefit from pancreatic surgery as well as adjuvant and palliative chemotherapy with acceptable morbidity [30, 31, 32]. Nevertheless, this group of patients needs to be even more carefully selected before being exposed to such treatments.

There has been a shift in the use of neoadjuvant treatment in Sweden during the last decade. According to our data it was rarely given in 2010–2011 (2.7%); its use increased to 9.1% in 2018–2019 with a further assumed rise in the years to come. Multiple studies investigating neoadjuvant therapies for both borderline and resectable tumours are ongoing which might result in a change in recommendations within the near future [19, 20, 33].

Among the resected patients there was no difference between the Early and Late groups regarding the number of patients receiving adjuvant therapy. Also, our data show that around 10% still do not recover sufficiently to start the adjuvant treatment within the recommended 8 weeks after surgery [4]. Adjuvant regimens used in 2010–2011 consisted mainly of gemcitabine as single therapy with a transition to GemCap in 2018–2019. In 2010 Neoptolemos et al. showed that gemcitabine alone was comparable to 5‐FU regarding its effect on survival but with fewer side effects and less toxicity [9]. Seven years later the same group demonstrated a survival benefit for GemCap, leading to a pan‐European change in adjuvant regimen [10]. In 2018, Conroy et al. [17] could present a median survival of 54.4 months in resected patients receiving mFOLFIRINOX as adjuvant treatment. An equal analysis is not possible to perform based on our material, as only three patients (all diagnosed in late 2019), were both resected and had mFOLFIRINOX as adjuvant treatment (Table S4). The current national Swedish recommendation for patients with superior PS is thus primarily mFOLFIRINOX and a further increase in the use of this treatment is likely to be seen in the upcoming years.

During the investigated period the use of first‐line chemotherapy seemed to increase, especially for the resected group (40.5% vs. 56.8%). Also, an adaptation towards combination therapies such as mFOLFIRINOX and especially GemNabP could be found, which is in line with the results of multiple studies implemented worldwide [8, 11, 32, 34, 35]. Rasmussen et al. [36] performed a nationwide study in Denmark comparing differences in chemotherapy regimens and survival between the years 2011–2013 and 2014–2016 [36]. Similar trends with higher frequencies of combination therapies and prolonged overall survival associated with mFOLFIRINOX were found. Still, the high level of toxicity prevents it from being administered to the common pancreatic cancer patient. Recent studies also indicate that GemNabP has an equal effect on survival when data are adjusted for differences in baseline characteristics [37, 38].

Despite a transition towards combination therapies no difference in overall survival was found for the palliative patients when comparing the Early and Late periods. However, this is a heterogeneous group of patients, from those receiving neoadjuvant treatment with curative intent to the group given only best supportive care. Also, our data show that the patients in the Late group of the palliative patients have a tendency to be older; still they seem to receive chemotherapy more frequently than the Early group. As such there might still be a survival benefit for the Late group due to the given therapy. Further sub‐investigation demands a much larger cohort of patients. In the resected group, despite a larger percentage of severe post‐operative complications, a significant improvement in 2‐year survival from 59.5% to 81.8% was found, also when data were adjusted in regression analysis. The difference is likely to be explained by multiple factors where novel chemotherapies, but also the introduction of an enhanced recovery program are important components [39]. Other possible influencers such as improved PS of the patients are less probable causes as patients of the Late group appeared to be both older and with more comorbidities.

Weaknesses of this study are its retrospective nature and the small number of patients both enrolled and treated in one single national region. Also, for the resected patients in the Late group, a longer follow‐up than 2 years would have been preferable as current data does not say anything about the consistency of the improved overall survival in this group. A strength is the detailed review of medical records making information solid and reliable. Also, all patients diagnosed with pancreatic adenocarcinoma within this specific region were included thus generating an unselected cohort describing real‐world data.

## 5 Conclusion

In summary, herein we present a thorough examination of chemotherapeutic regimens and survival rates of patients diagnosed with pancreatic cancer in 2010–2011 and 2018–2019 in the southern region of Sweden. Main findings are a gradual change towards combination therapies such as mFOLFIRINOX, GemCap and GemNabP, thus following both global and national recommendations for palliative and resected patients. Additionally, an improvement in 2‐year overall survival was demonstrated for the resected group. However, further follow‐up is needed to determine whether this development is persistent.

## Author Contributions

**Hedda Tranvik :** conceptualisation (equal), data curation (equal), formal analysis (equal), investigation (equal), methodology (equal), project administration (equal), writing – original draft (equal). **Farima Brandt:** conceptualisation (supporting), methodology (supporting), validation (equal), visualisation (equal), writing – review and editing (equal). **Caroline Williamsson:** data curation (supporting), formal analysis (supporting), methodology (supporting), validation (supporting), writing – review and editing (equal). **Hanna Sternby:** conceptualisation (equal), formal analysis (equal), funding acquisition (lead), investigation (equal), methodology (equal), project administration (equal), resources (lead), supervision (lead), validation (equal), writing – original draft (equal), writing – review and editing (lead).

## Funding

The authors have nothing to report.

## Ethics Statement

Ethical approval was obtained from the Swedish National Board of Ethical Decisions, number 2020‐01402.

## Consent

Through the Swedish National Pancreatic and Periampullary Cancer Registry informed consent has been obtained from all included patients. No identifying information about the participants was available from the registry data.

## Conflicts of Interest

The authors declare no conflicts of interest.

## Supporting information


**Table S1:** TNM‐stage palliative patients.
**Table S2:** Detailed chemotherapy regimens for palliative patients.
**Table S3:** T‐ and N‐stage and UICC‐stage for resected patients.
**Table S4:** Detailed chemotherapy regimens for resected patients.
**Figure S1:** Consort diagram of patient inclusion.

## Data Availability

The data that support the findings of this study are available on request from the corresponding author. The data are not publicly available due to privacy or ethical restrictions.

## References

[1] G. Engholm , M. Gislum , F. Bray , and T. Hakulinen , “Trends in the Survival of Patients Diagnosed With Cancer in the Nordic Countries 1964–2003 Followed Up to the End of 2006. Material and Methods,” Acta Oncologica 49 (2010): 545–560. 20491523 10.3109/02841861003739322

[2] Nordcan , “Association of the Nordic Cancer Registries,” https://nordcan.iarc.fr/en.

[3] M. H. Katz , H. Wang , J. B. Fleming , et al., “Long‐Term Survival After Multidisciplinary Management of Resected Pancreatic Adenocarcinoma,” Annals of Surgical Oncology 16 (2009): 836–847. 19194760 10.1245/s10434-008-0295-2 PMC3066077

[4] S. J. Ma , O. T. Oladeru , J. A. Miccio , A. J. Iovoli , G. M. Hermann , and A. K. Singh , “Association of Timing of Adjuvant Therapy With Survival in Patients With Resected Stage I to II Pancreatic Cancer,” JAMA Network Open 2 (2019): e199126. 31411712 10.1001/jamanetworkopen.2019.9126 PMC6694394

[5] H. Oettle , P. Neuhaus , A. Hochhaus , et al., “Adjuvant Chemotherapy With Gemcitabine and Long‐Term Outcomes Among Patients With Resected Pancreatic Cancer: The CONKO‐001 Randomized Trial,” JAMA 310 (2013): 1473–1481. 24104372 10.1001/jama.2013.279201

[6] H. A. Burris, 3rd , M. J. Moore , J. Andersen , et al., “Improvements in Survival and Clinical Benefit With Gemcitabine as First‐Line Therapy for Patients With Advanced Pancreas Cancer: A Randomized Trial,” Journal of Clinical Oncology 15 (1997): 2403–2413. 9196156 10.1200/JCO.1997.15.6.2403

[7] M. J. Moore , D. Goldstein , J. Hamm , et al., “Erlotinib Plus Gemcitabine Compared With Gemcitabine Alone in Patients With Advanced Pancreatic Cancer: A Phase Iii Trial of the National Cancer Institute of Canada Clinical Trials Group,” Journal of Clinical Oncology 25 (2007): 1960–1966. 17452677 10.1200/JCO.2006.07.9525

[8] D. D. Von Hoff , T. Ervin , F. P. Arena , et al., “Increased Survival in Pancreatic Cancer With Nab‐Paclitaxel Plus Gemcitabine,” New England Journal of Medicine 369 (2013): 1691–1703. 24131140 10.1056/NEJMoa1304369 PMC4631139

[9] J. P. Neoptolemos , D. D. Stocken , C. Bassi , et al., “Adjuvant Chemotherapy With Fluorouracil Plus Folinic Acid vs Gemcitabine Following Pancreatic Cancer Resection: A Randomized Controlled Trial,” JAMA 304 (2010): 1073–1081. 20823433 10.1001/jama.2010.1275

[10] J. P. Neoptolemos , D. H. Palmer , P. Ghaneh , et al., “Comparison of Adjuvant Gemcitabine and Capecitabine With Gemcitabine Monotherapy in Patients With Resected Pancreatic Cancer (ESPAC‐4): A Multicentre, Open‐Label, Randomised, Phase 3 Trial,” Lancet 389 (2017): 1011–1024. 28129987 10.1016/S0140-6736(16)32409-6

[11] T. Conroy , F. Desseigne , M. Ychou , et al., “Folfirinox Versus Gemcitabine for Metastatic Pancreatic Cancer,” New England Journal of Medicine 364 (2011): 1817–1825. 21561347 10.1056/NEJMoa1011923

[12] H. Mahaseth , E. Brutcher , J. Kauh , et al., “Modified Folfirinox Regimen With Improved Safety and Maintained Efficacy in Pancreatic Adenocarcinoma,” Pancreas 42 (2013): 1311–1315. 24152956 10.1097/MPA.0b013e31829e2006

[13] H. Tong , Z. Fan , B. Liu , and T. Lu , “The Benefits of Modified Folfirinox for Advanced Pancreatic Cancer and Its Induced Adverse Events: A Systematic Review and Meta‐Analysis,” Scientific Reports 8 (2018): 8666. 29875415 10.1038/s41598-018-26811-9 PMC5989209

[14] H. Oettle , H. Riess , J. M. Stieler , et al., “Second‐Line Oxaliplatin, Folinic Acid, and Fluorouracil Versus Folinic Acid and Fluorouracil Alone for Gemcitabine‐Refractory Pancreatic Cancer: Outcomes From the CONKO‐003 Trial,” Journal of Clinical Oncology 32 (2014): 2423–2429. 24982456 10.1200/JCO.2013.53.6995

[15] Kunskapsbanken Cancercentrum , “National Guidelines for Pancreatic Cancer Care,” Sweden, (2024), https://kunskapsbanken.cancercentrum.se/diagnoser/bukspottkortelcancer/vardprogram/.

[16] M. M. Oken , R. H. Creech , D. C. Tormey , et al., “Toxicity and Response Criteria of the Eastern Cooperative Oncology Group,” American Journal of Clinical Oncology 5 (1982): 649–655. 7165009

[17] T. Conroy , P. Hammel , M. Hebbar , et al., “Folfirinox or Gemcitabine as Adjuvant Therapy for Pancreatic Cancer,” New England Journal of Medicine 379 (2018): 2395–2406. 30575490 10.1056/NEJMoa1809775

[18] M. M. Assifi , X. Lu , G. Eibl , H. A. Reber , G. Li , and O. J. Hines , “Neoadjuvant Therapy in Pancreatic Adenocarcinoma: A Meta‐Analysis of Phase II Trials,” Surgery 150 (2011): 466–473. 21878232 10.1016/j.surg.2011.07.006 PMC3164966

[19] K. J. Labori , S. O. Bratlie , B. Andersson , et al., “Neoadjuvant FOLFIRINOX Versus Upfront Surgery for Resectable Pancreatic Head Cancer (NORPACT‐1): A Multicentre, Randomised, Phase 2 Trial,” Lancet Gastroenterology & Hepatology 9 (2024): 205–217. 38237621 10.1016/S2468-1253(23)00405-3

[20] J. L. van Dam , E. M. M. Verkolf , E. N. Dekker , et al., “Perioperative or Adjuvant mFOLFIRINOX for Resectable Pancreatic Cancer (PREOPANC‐3): Study Protocol for a Multicenter Randomized Controlled Trial,” BMC Cancer 23 (2023): 728. 37550634 10.1186/s12885-023-11141-5 PMC10405377

[21] D. Cunningham , I. Chau , D. D. Stocken , et al., “Phase III Randomized Comparison of Gemcitabine Versus Gemcitabine Plus Capecitabine in Patients With Advanced Pancreatic Cancer,” Journal of Clinical Oncology 27 (2009): 5513–5518. 19858379 10.1200/JCO.2009.24.2446

[22] J. H. Kim , S. C. Lee , S. Y. Oh , et al., “Attenuated FOLFIRINOX in the Salvage Treatment of Gemcitabine‐Refractory Advanced Pancreatic Cancer: A Phase ii Study,” Cancer Communications 38 (2018): 32. 29866170 10.1186/s40880-018-0304-1 PMC5993129

[23] S. Gill , Y. J. Ko , C. Cripps , et al., “PANCREOX: A Randomized Phase Iii Study of Fluorouracil/Leucovorin With or Without Oxaliplatin for Second‐Line Advanced Pancreatic Cancer in Patients Who Have Received Gemcitabine‐Based Chemotherapy,” Journal of Clinical Oncology 34 (2016): 3914–3920. 27621395 10.1200/JCO.2016.68.5776

[24] K. J. Roberts , H. Schrem , J. Hodson , et al., “Pancreas Exocrine Replacement Therapy Is Associated With Increased Survival Following Pancreatoduodenectomy for Periampullary Malignancy,” HPB 19 (2017): 859–867. 28711377 10.1016/j.hpb.2017.05.009

[25] K. J. Roberts , C. A. Bannister , and H. Schrem , “Enzyme Replacement Improves Survival Among Patients With Pancreatic Cancer: Results of a Population Based Study,” Pancreatology 19 (2019): 114–121. 30385188 10.1016/j.pan.2018.10.010

[26] J. H. Jung , S. H. Won , K. Jung , et al., “Analysis of Recent Improvement of Survival Outcomes in Patients With Pancreatic Cancer Who Underwent Upfront Surgery,” Gut Liver 18, no. 4 (2024): 737–746. 38146258 10.5009/gnl230303 PMC11249928

[27] T. F. Stoop , A. A. Javed , A. Oba , et al., “Pancreatic Cancer,” Lancet 405, no. 10485 (2025): 1182–1202. 40187844 10.1016/S0140-6736(25)00261-2

[28] A. D. Parmar , G. M. Vargas , N. P. Tamirisa , K. M. Sheffield , and T. S. Riall , “Trajectory of Care and Use of Multimodality Therapy in Older Patients With Pancreatic Adenocarcinoma,” Surgery 156 (2014): 280–289. 24851723 10.1016/j.surg.2014.03.001 PMC4099282

[29] O. Higuera , I. Ghanem , R. Nasimi , I. Prieto , L. Koren , and J. Feliu , “Management of Pancreatic Cancer in the Elderly,” World Journal of Gastroenterology 22 (2016): 764–775. 26811623 10.3748/wjg.v22.i2.764 PMC4716075

[30] H. M. Park , S. J. Park , S. S. Han , and S. H. Kim , “Surgery for Elderly Patients With Resectable Pancreatic Cancer, a Comparison With Non‐Surgical Treatments: A Retrospective Study Outcomes of Resectable Pancreatic Cancer,” BMC Cancer 19 (2019): 1090. 31718565 10.1186/s12885-019-6255-3 PMC6852721

[31] M. Macchini , M. Chiaravalli , S. Zanon , et al., “Chemotherapy in Elderly Patients With Pancreatic Cancer: Efficacy, Feasibility and Future Perspectives,” Cancer Treatment Reviews 72 (2019): 1–6. 30414985 10.1016/j.ctrv.2018.10.013

[32] G. W. Prager , L. Oehler , A. Gerger , et al., “Comparison of Nab‐Paclitaxel Plus Gemcitabine in Elderly Versus Younger Patients With Metastatic Pancreatic Cancer: Analysis of a Multicentre, Prospective, Non‐Interventional Study,” European Journal of Cancer 143 (2021): 101–112. 33296830 10.1016/j.ejca.2020.11.003

[33] Q. P. Janssen , J. L. van Dam , B. A. Bonsing , et al., “Total Neoadjuvant FOLFIRINOX Versus Neoadjuvant Gemcitabine‐Based Chemoradiotherapy and Adjuvant Gemcitabine for Resectable and Borderline Resectable Pancreatic Cancer (PREOPANC‐2 Trial): Study Protocol for a Nationwide Multicenter Randomized Controlled Trial,” BMC Cancer 21 (2021): 300. 33757440 10.1186/s12885-021-08031-z PMC7989075

[34] H. Blomstrand , U. Scheibling , C. Bratthall , H. Green , and N. O. Elander , “Real World Evidence on Gemcitabine and Nab‐Paclitaxel Combination Chemotherapy in Advanced Pancreatic Cancer,” BMC Cancer 19 (2019): 40. 30621618 10.1186/s12885-018-5244-2 PMC6325739

[35] Y. Wang , P. Camateros , and W. Y. Cheung , “A Real‐World Comparison of FOLFIRINOX, Gemcitabine Plus Nab‐Paclitaxel, and Gemcitabine in Advanced Pancreatic Cancers,” Journal of Gastrointestinal Cancer 50 (2019): 62–68. 29143916 10.1007/s12029-017-0028-5

[36] L. S. Rasmussen , C. W. Fristrup , B. V. Jensen , et al., “Initial Treatment and Survival in 4163 Danish Patients With Pancreatic Cancer: A Nationwide Unselected Real‐World Register Study,” European Journal of Cancer 129 (2020): 50–59. 32120275 10.1016/j.ejca.2020.01.015

[37] J. M. Riedl , F. Posch , L. Horvath , et al., “Gemcitabine/Nab‐Paclitaxel Versus FOLFIRINOX for Palliative First‐Line Treatment of Advanced Pancreatic Cancer: A Propensity Score Analysis,” European Journal of Cancer 151 (2021): 3–13. 33951545 10.1016/j.ejca.2021.03.040

[38] S. Kim , J. E. Signorovitch , H. Yang , et al., “Comparative Effectiveness of Nab‐Paclitaxel Plus Gemcitabine vs FOLFIRINOX in Metastatic Pancreatic Cancer: A Retrospective Nationwide Chart Review in the United States,” Advances in Therapy 35 (2018): 1564–1577. 30209750 10.1007/s12325-018-0784-z PMC6182639

[39] C. Williamsson , N. Karlsson , C. Sturesson , G. Lindell , R. Andersson , and B. Tingstedt , “Impact of a Fast‐Track Surgery Programme for Pancreaticoduodenectomy,” British Journal of Surgery 102 (2015): 1133–1141. 26042725 10.1002/bjs.9856

