# Supplementary material for: Chemotherapy Regimens and Survival in Pancreatic Cancer—A Ten‐Year Single Centre Overview
Source: Cancer Med. 2025 Dec 2;14(23):e71416. doi: 10.1002/cam4.71416 (PMC12672207; doi:10.1002/cam4.71416)

**Supplements**

Table S1

TNM-stage palliative patients

|  | 2010-2011  n (%) | 2018-2019  n (%) | p-value |
| --- | --- | --- | --- |
| T-stage | | | |
| TX | 20 (23) | 13 (15) | 0.24 |
| T1 | 1 | 0 | 0.27 |
| T2 | 10 (11) | 9 (11) | 0.51 |
| T3 | 31 (35) | 35 (41) | 0.77 |
| T4 | 27 (30) | 29 (34) | 0.64 |
| N-stage |  |  |  |
| NX | 73 (66) | 70 (53) | 0.04 |
| N0 | 7 (6) | 12 (9) | 0.43 |
| N1 | 30 (27) | 50 (38) | 0.08 |
| M-stage |  |  |  |
| M0 | 75 (68) | 70 (53) | 0.03 |
| M1 | 35 (32) | 62 (47) | 0.02 |

TX; not possible to define T-stage, NX; not possible to define N-stage

Table S2

Detailed chemotherapy regimens for palliative patients

|  | | 2010-2011  n (%) | 2018-2019  n (%) | p-value |
| --- | --- | --- | --- | --- |
| Neoadjuvant treatment | No chemotherapy | 106 (96) | 118 (89) | 0.04 |
|  | mFOLFIRINOX | 3 (3) | 13 (10) | 0.03 |
|  | Gemcitabine | 1 (1) | 0 (0) | 0.46 |
|  | GemNabP | 0 (0) | 1 (1) | 1.00 |
| First line treatment | No chemotherapy | 48 (44) | 46 (35) | 0.16 |
|  | mFOLFIRINOX | 1 (1) | 23 (17) | <0.001 |
|  | Gemcitabine | 49 (45) | 23 (17) | <0.001 |
|  | GemNabP | 1 (1) | 38 (30) | <0.001 |
|  | GemCap | 10 (9) | 0 (0) | <0.001 |
|  | FLOX | 1 (1) | 2 (2) | 1.00 |
| Second line treatment | No chemotherapy | 92 (84) | 94 (71) | 0.02 |
|  | Gemcitabine | 2 (2) | 9 (7) | 0.06 |
|  | GemNabP | 0 (0) | 11 (8) | 0.002 |
|  | GemCap | 5 (5) | 0 (0) | 0.02 |
|  | FLOX | 11 (10) | 10 (8) | 0.50 |
|  | FLV | 0 (0) | 2 (2) | 0.50 |
|  | Tegafur | 0 (0) | 5 (4) | 0.07 |
|  | SOX | 0 (0) | 1 (1) | 1.00 |

Shown as n (%); mFOLFIRINOX, modified 5-fluorouracil+leucovorin+irinotecan+oxaliplatin; GemNabP, gemcitabine+ nabPaclitaxel; GemCap, gemcitabine+capecetabine; FLOX, fluorouracil+ oxaliplatin; FLV, fluorouracil; SOX, oxaliplatin

Table S3

*T- and N-stage and UICC-stage for resected patients*

|  | 2010-2011  n (%) | 2018-2019  n (%) | p-value |
| --- | --- | --- | --- |
| T-status |  |  |  |
| T1 | 4 (10.8) | 1 (2.3) | 0.11 |
| T2 | 8 (21.6) | 18 (40.9) | 0.07 |
| T3 | 22 (59.5) | 22 (50.0) | 0.40 |
| T4 | 3 (8.1) | 3 (6.8) | 0.83 |
| N-status |  |  |  |
| N0 | 10 (26.6) | 9 (20.5) | 0.41 |
| N1 | 25 (71.4) | 35 (79.5) | 0.23 |
| UICC-stage |  |  |  |
| IA | 2 (5.4) | 1 (2.3) |  |
| IB | 3 (8.1) | 3 (6.8) |  |
| IIA | 5 (13.5) | 5 (11.3) |  |
| IIB | 15 (40.5) | 15 (34.1) |  |
| III | 12 (32.4) | 20 (45.5) |  |
| IV | 0 | 0 | 0.51 |

No metastases (M-stage) where found within the resected group

UICC; Union for International Cancer Control

Table S4

Detailed chemotherapy regimens for resected patients

|  | | 2010-2011  n (%) | 2018-2019  n (%) | p-value |
| --- | --- | --- | --- | --- |
| Neoadjuvant treatment | No chemotherapy | 36 (97.3) | 40 (90.9) | 0.37 |
|  | mFOLFIRINOX | 1 (2.7) | 3 (6.8) | 0.62 |
|  | GemNabP | 0 (0.0) | 1 (2.3) | 1.00 |
| Adjuvant treatment | No chemotherapy | 4 (10.8) | 8 (18.2) | 0.53 |
|  | mFOLFIRINOX | 0 (0.0) | 3 (6.8) | 0.246 |
|  | Gemcitabine | 30 (81.1) | 10 (22.7) | <0.001 |
|  | GemCap | 3 (8.1) | 22 (50.0) | <0.001 |
|  | Capecetabin | 0 (0.0) | 1 (2.3) | 1.00 |
| First line treatment | No chemotherapy | 22 (59.5) | 19 (43.2) | 0.18 |
|  | mFOLFIRINOX | 2 (5.4) | 7 (15.9) | 0.15 |
|  | Gemcitabine | 8 (21.6) | 3 (6.8) | 0.10 |
|  | GemNabP | 0 (0.0) | 9 (20.5) | 0.004 |
|  | GemCap | 3 (8.1) | 0 (0.0) | 0.06 |
|  | FLOX | 1 (2.7) | 5 (11.4) | 0.21 |
|  | FLV | 1 (2.7) | 1 (2.3) | 1.00 |
|  | SOX | 0 (0.0) | 1 (2.3) | 1.00 |
| Second line treatment | No chemotherapy | 31 (83.8) | 31 (70.5) | 0.16 |
|  | Gemcitabine | 1 (2.7) | 2 (4.5) | 1.00 |
|  | GemNabP | 0 (0.0) | 3 (6.8) | 0.25 |
|  | FLOX | 5 (13.5) | 0 (0.0) | 0.01 |
|  | Tegafur | 0 (0.0) | 6 (13.6) | 0.02 |
|  | CAPOX | 0 (0.0) | 1 (2.3) | 1.00 |

Shown as n (%); mFOLFIRINOX, modfied 5-fluorouracil+leucovorin+irinotecan+oxaliplatin; GemNabP, gemcitabine+ nabPaclitaxel; GemCap, gemcitabine+capecetabine; FLOX, fluorouracil+ oxaliplatin; FLV, fluorouracil; SOX, oxaliplatin; CAPOX, oxaliplatin+ capecetabine

Supplements Figure 1

*Consort diagram of patient inclusion*


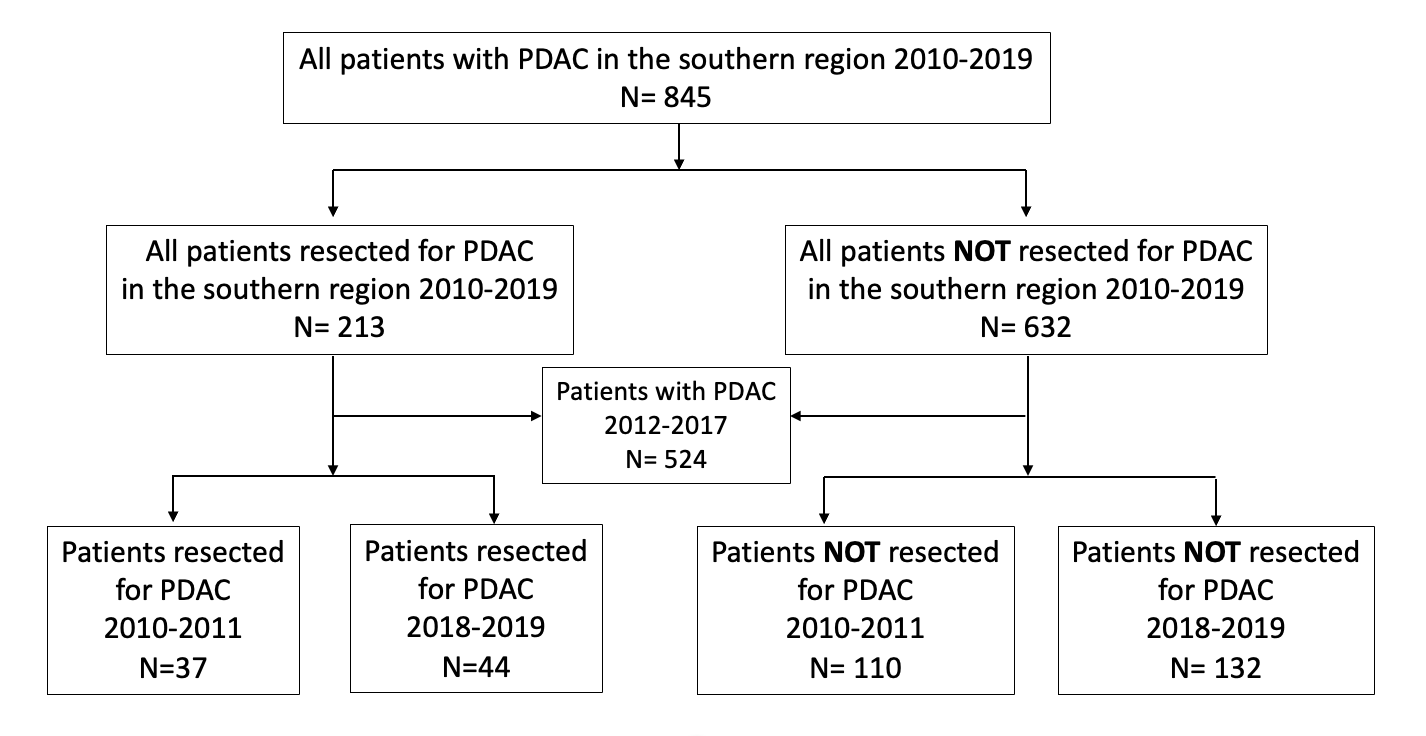

Supplement: Supplementary file 1 — Table S1: TNM‐stage palliative patients. Table S2: Detailed chemotherapy regimens for palliative patients. Table S3: T‐ and N‐stage and UICC‐stage for resected patients. Table S4: Detailed chemotherapy regimens for resected patients. Figure S1: Consort diagram of patient inclusion. [file CAM4-14-e71416-s001.docx]
